# A combined 3D-QSAR and docking studies for the *In-silico* prediction of HIV-protease inhibitors

**DOI:** 10.1186/1752-153X-7-88

**Published:** 2013-05-17

**Authors:** Zaheer Ul-Haq, Saman Usmani, Hina Shamshad, Uzma Mahmood, Sobia Ahsan Halim

**Affiliations:** 1 Dr. Panjwani Center for Molecular Medicine and Drug Research, International Center for Chemical and Biological Sciences, University of Karachi, Karachi 75270, Pakistan

**Keywords:** HIV-PIs, AIDs, CoMFA, CoMSIA, 3D-QSAR, GOLD

## Abstract

**Background:**

Tremendous research from last twenty years has been pursued to cure human life against HIV virus. A large number of HIV protease inhibitors are in clinical trials but still it is an interesting target for researchers due to the viral ability to get mutated. Mutated viral strains led the drug ineffective but still used to increase the life span of HIV patients.

**Results:**

In the present work, 3D-QSAR and docking studies were performed on a series of Danuravir derivatives, the most potent HIV- protease inhibitor known so far. Combined study of 3D-QSAR was applied for Danuravir derivatives using ligand-based and receptor-based protocols and generated models were compared. The results were in good agreement with the experimental results. Additionally, docking analysis of most active 32 and least active 46 compounds into wild type and mutated protein structures further verified our results. The 3D-QSAR and docking results revealed that compound 32 bind efficiently to the wild and mutated protein whereas, sufficient interactions were lost in compound 46.

**Conclusion:**

The combination of two computational techniques would helped to make a clear decision that compound 32 with well inhibitory activity bind more efficiently within the binding pocket even in case of mutant virus whereas compound 46 lost its interactions on mutation and marked as least active compound of the series. This is all due to the presence or absence of substituents on core structure, evaluated by 3D-QSAR studies. This set of information could be used to design highly potent drug candidates for both wild and mutated form of viruses.

## Background

Human immunodeficiency virus (HIV) is a retrovirus that is peril to human health, responsible to cause AIDS, an immunodeficiency syndrome. The disease presents a serious health care challenge because each year it affects an increasing number of people across the globe [1]. To combat disease, several new drugs were approved by FDA which reduces the morbidity and mortality of HIV infection. These drugs are categorized as HIV-Reverse transcriptase (HIV-RT), HIV-Integrase (HIN-IN) & HIV-Protease inhibitors (HIV-PIs), the major targeted enzymes of HIV life cycle. HAART (highly active anti-retroviral therapy) is the most promising anti-AIDS therapy including these inhibitors in combination. The major obstacle in the use of HAART therapy is resistance that virus develops [2]. The hyper-mutability of HIV, drug resistance and their side effects are the biggest challenge to develop an effective anti-AIDS therapy.

HIV-1 Protease is emerging as one of the major druggable target for the development of new chemotherapeutics. HIV protease inhibitors, restrain the viral maturation by preventing the formation of structural and functional proteins and form immature, non-infectious virus. However, it is highly prone to develop mutations, since it is a homodimer and a single mutation of gene causes double mutation of enzyme [3]. Structurally, HIV protease is a homodimer protein, containing 99 amino acids in each chain, with an active site located at the dimer interface [4]. The protein is composed of three regions; catalytic core (Asp25, Gly27, Ala28, Asp29 and Asp30), flap (Ile47, Gly48, Gly49, and Ile50) and the C-terminal region (Pro81, and Ile84). From literature, Asp25, Gly27, Ala28, Asp29 and Gly49 are known to be highly conserved residues to which a potent inhibitor may bind strongly. Mutations of HIV protease at Val32, Ile50 and Ile84 (hydrophobic residues, close to binding pocket) are responsible for the resistance to most FDA approved drugs due to loss of Vander Waal interactions [5]. Almost all FDA approved anti-AIDs drugs are resistant to I84V mutant virus and became ineffective against disease.

The failure of drug therapies against mutated virus protein encouraged the scientists to develop more potent, effective and stable second generation HIV-PIs, but still the HIV-PI therapies are associated with the serious problems that limit their significance and effectiveness [6]. In order to take a forward step for prediction and guidance of more effective drug, 3D-QSAR studies were conducted as primitive step in finding new inhibitors using a dataset of 102 *(R)-hydroxyethylamino sulfonamides* derivatives from literature [7].

3D-QSAR technique is subdivided into ligand-based and structure-based methods. Ligand-based approach is frequently applicable in the absence of experimentally resolved protein crystal structure whereas, structure-based method extract the protein bound ligand information for the generation of align model [8-10]. In the present work, both strategies were applied to generate the CoMFA and CoMSIA models and their comparison with reference to the most active moiety Darunavir (hydroxyethylamino sulfonamides derivatives). Extensive research is ongoing that used different scaffolds, methodology and algorithms for predicting better results. Darunavir (DRV) is one of the most attracting targets as it is the most active molecule among eleven FDA approved drugs of present time [11]. The obtained models revealed the significance of stereoelectronic properties, hydrogen bonding characteristics and structure variations leading to changes in the interaction profile. The influences of grid distances, alignment methods and combination of charges were explored out of which the best model was selected. Additionally, molecular docking of compounds explored the binding affinity of highly active and least active compounds with its receptor by using GOLD docking suit [12]. The purpose of the study was to validate the experimental results obtained with Darunavir derivatives and to predict the compound that may developed into a more potent HIV inhibitor based on outcomes extracted from the current study.

## Results and discussion

Protease active site is composed of catalytic triad having two C2 symmetrical monomeric units, Asp25 (25')-Thr26 (26')-Gly27 (27'). This triad is surrounded by amino-acids, classified into S1 (1') and S2 (2') sub-sites, which mostly include the hydrophobic amino-acids [13]. However, on ligand binding, Protease behaves as asymmetrical monomer [14]. Darunavir, an FDA approved drug has shown extensive hydrogen bonding with protease backbone, especially with S2 sub-site of protease, moreover it also retained interaction with mutated protein [15].

In the present work, the additive model of *Jorissen R.N. et.al.*, [7] was further subjected to 3D-QSAR using CoMFA & CoMSIA techniques and the generated contour maps were further validated by molecular docking.

### Statistics of the ligand-based models

**Figure 1:**
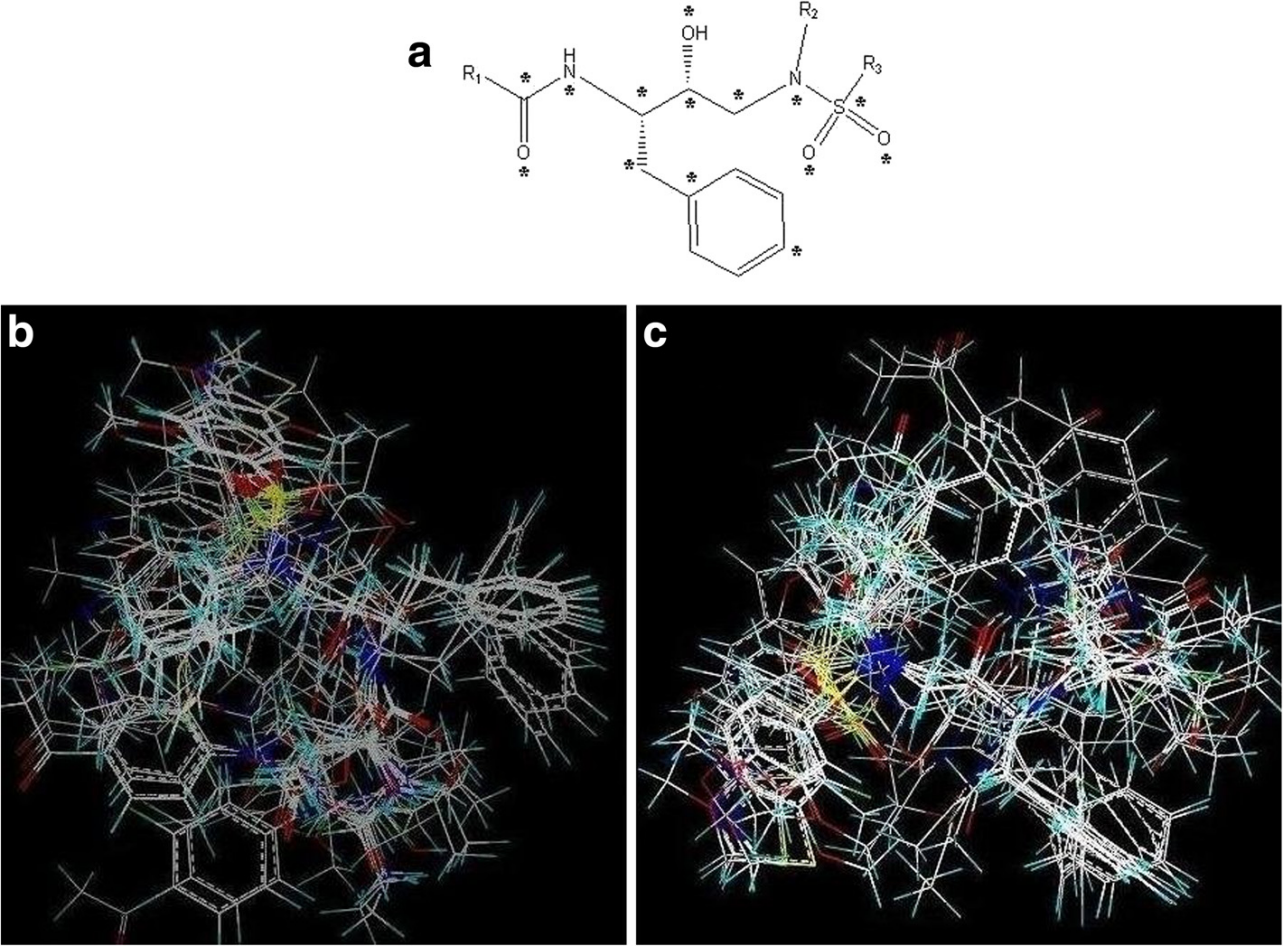
**Core structure and dataset alignment. a**) Core structure of danuravir derivatives with marked points used for alignment, **b**) Ligand-based alignment by using most active compound 32 as template, **c**) Structure-based alignment using cognate ligand of 3QOZ.pdb as reference.

The reliability of CoMFA and CoMSIA models were highly dependent on the better alignment of molecules in a three dimensional space. The database alignment implemented in Sybyl7.3 [16] was used to align 102 compounds using most active compound 32 as a template. The core structure of compound 32 was chosen as a structural element for superimposition of all other compounds (Figure 1a). The alignment is shown in Figure 1b and c. The statistical model of training and test tests (Tables 1 and 2) generated for the initial data set was depicted in Table 3. From the results, it can be deduced that lowering the grid space showed negative impact on the model. The default value of the grid space was selected as best and was used for further studies. To validate the model by external test set, activities of 24 compounds were predicted and the residual values for external and internal data sets were evaluated (Table 2). The best model with convincing statistical results is shown in Table 3 and the residual value for the best model was found to be less than 1 in both training and test sets as mentioned in Tables 1 and 2. Furthermore CoMSIA was applied on the same dataset and the results are tabulated in Table 4.

**Table 1:** **Ligand-based and structure-based, actual and predicted pIC**_**50 **_**values of training set generated by CoMFA model along with their residuals**

**Compounds**	**pIC**_**50**_	**Ligand-based**		**Structure-based**	
		**Predicted**	**Residuals**	**Predicted**	**Residuals**
Comp 004	6.62	6.51	-0.11	6.71	-0.09
Comp 005	6.77	7.06	0.29	7.31	-0.54
Comp 006	7.38	6.78	-0.6	6.94	0.44
Comp 007	10.08	10.37	0.29	10.09	-0.01
Comp 008	9.77	9.7	-0.07	9.83	-0.06
Comp 009	10.15	10.13	-0.02	9.95	0.20
Comp 011	6.72	6.45	-0.27	6.64	0.09
Comp 012	6.8	7	0.2	7.23	-0.43
Comp 014	9.59	10.08	0.49	9.63	-0.04
Comp 015	11.4	10.63	-0.77	10.32	1.09
Comp 016	9.08	9.26	0.18	9.23	-0.15
Comp 017	9.74	9.95	0.21	10.05	-0.31
Comp 018	10.1	10.4	0.3	10.19	-0.09
Comp 019	10.8	10.44	-0.36	10.73	0.08
Comp 020	7.53	7.92	0.38	7.82	-0.29
Comp 021	6.78	7.19	0.41	7.33	-0.55
Comp 022	9.1	9.2	0.1	9.19	-0.09
Comp 023	10.18	10.07	-0.12	9.95	0.23
Comp 025	9.46	9.82	0.36	9.82	-0.36
Comp 026	10.07	9.86	-0.21	10.36	-0.29
Comp 027	9.42	9.73	0.31	9.59	-0.17
Comp 029	10.38	10.17	-0.21	10.55	-0.16
Comp 030	10.14	10.6	0.46	10.68	-0.54
Comp 031	10.8	10.62	-0.18	10.73	0.07
Comp 032	12.1	11.345	-0.75	11.037	1.06
Comp 033	10.49	10.78	0.29	10.89	-0.40
Comp 034	11.22	11.23	0.01	11.08	0.14
Comp 035	9.63	9.47	-0.17	9.41	0.22
Comp 036	10.72	10.68	-0.04	10.17	0.55
Comp 037	9.93	9.54	-0.39	9.78	0.15
Comp 038	7.48	7.21	-0.27	7.02	0.46
Comp 040	5.97	6.36	0.39	6.44	-0.47
Comp 041	9.41	9.46	0.05	9.32	0.09
Comp 042	7.28	6.46	-0.82	6.51	0.77
Comp 043	4.88	5.526	0.65	5.396	-0.52
Comp 044	9.77	9.208	-0.56	9.026	0.74
Comp 045	6.21	6.219	0.01	6.21	0
Comp 046	4.58	5.291	0.71	5.097	-0.52
Comp 047	5.63	6.211	0.58	6.33	-0.7
Comp 048	6.29	6.138	-0.15	6.334	-0.04
Comp 049	10.03	10.297	0.27	10.11	-0.08
Comp 050	7.39	6.763	-0.63	6.65	0.74
Comp 051	10.48	10.46	-0.02	10.28	0.2
Comp 052	7.3	6.22	-1.08	6.42	0.88
Comp 053	9.42	10.15	0.73	10.1	-0.68
Comp 056	7.62	6.93	-0.69	6.78	0.84
Comp 057	7.24	7.26	0.02	6.97	0.27
Comp 058	5.93	6.59	0.66	6.73	-0.80
Comp 060	6.27	6.57	0.3	6.45	-0.18
Comp 061	4.84	4.35	-0.49	4.44	0.40
Comp 063	4.91	4.58	-0.33	4.31	0.60
Comp 066	5.69	6.39	0.7	6.34	-0.65
Comp 069	4.87	5.65	0.78	5.59	-0.72
Comp 070	6.59	6.05	-0.54	6.05	0.54
Comp 071	9.62	9.56	-0.07	9.33	0.29
Comp 072	9.92	10.03	0.11	10.23	-0.30
Comp 073	8.38	8.32	-0.06	8.28	0.10
Comp 075	10.21	10.09	-0.12	9.75	0.46
Comp 077	9.85	9.94	0.09	10.04	-0.19
Comp 078	10.57	10	-0.57	9.89	0.68
Comp 079	8.84	9.33	0.49	9.38	-0.54
Comp 080	9.51	9.26	-0.25	9.44	0.07
Comp 081	9.93	10.21	0.28	10	-0.07
Comp 085	10.42	10.41	-0.01	10.47	-0.05
Comp 086	10.85	10.1	-0.75	10.16	0.69
Comp 088	10.24	10.59	0.35	11.04	-0.80
Comp 089	8.73	9.53	0.8	9.47	-0.74
Comp 091	8.61	8.74	0.13	9.19	-0.58
Comp 092	9.09	8.79	-0.3	9.04	0.05
Comp 094	10.2	9.41	-0.79	9.94	0.26
Comp 095	9.76	9.76	0	9.75	0.01
Comp 096	9.94	9.82	-0.12	9.61	0.33
Comp 098	9.68	9.54	-0.14	9.18	0.50
Comp 100	9.88	9.7	-0.18	9.78	0.10
Comp 101	9.43	9.93	0.5	10.06	-0.63
Comp 102	9.87	9.98	0.11	9.92	-0.05
Comp 104	9.15	9.2	0.05	9.65	-0.50
Comp 106	10.17	10.35	0.18	10.18	0.00

**Table 2:** **Ligand-based and structure-based, actual and predicted pIC**_**50 **_**values of test set generated by CoMFA model along with their residuals**

**Compounds**	**pIC**_**50**_	**Ligand-based model**		**Structure-based model**	
		**Predicted**	**Residuals**	**Predicted**	**Residuals**
Comp 001	10	10.24	0.24	10.02	-0.02
Comp 002	8.42	8.87	0.45	8.92	-0.5
Comp 003	9.28	9.56	0.28	9.75	-0.47
Comp 010	9.97	10.17	0.2	10.5	-0.53
Comp 013	6.82	6.74	-0.08	6.89	-0.07
Comp 024	9.64	9.39	-0.25	9.69	-0.05
Comp 028	11.22	10.74	-0.48	10.69	0.53
Comp 039	10.34	10.57	0.23	10.08	0.26
Comp 054	5.94	6.62	0.68	6.69	-0.75
Comp 055	6.23	6.44	0.21	6.76	-0.53
Comp 059	6.92	6.18	-0.75	5.98	0.94
Comp 064	5.32	4.88	-0.44	6.28	-0.96
Comp 074	8.79	8.43	-0.36	8.39	0.4
Comp 076	10.2	10.04	-0.16	10.17	0.03
Comp 082	10.44	10.27	-0.17	9.86	0.58
Comp 083	10.08	10.82	0.74	10.58	-0.5
Comp 084	10	10.87	0.87	10.44	-0.44
Comp 087	10.48	10.13	-0.35	10.14	0.34
Comp 090	9.54	9.58	0.04	9.33	0.21
Comp 093	9.21	8.95	-0.26	9.03	0.18
Comp 097	9.1	9.65	0.55	9.12	-0.02
Comp 099	9.68	9.39	-0.29	9.04	0.64
Comp 103	9.07	9.26	0.19	9.35	-0.28
Comp 105	9.47	10.3	0.83	10.31	-0.84

**Table 3:** The statistics of all generated CoMFA models in order to obtained the best model

**Charges**	**Model**	**GS**	**q**^**2**^	**SEP**	**C**	**r**^**2**^	**SEE**	**F**	$r_{\mathrm{pred}}^{2}$
**Ligand-Based Method**									
**Gasteiger Huckel**	**First**	0.5	0.77	0.9	6	0.93	0.45	171.85	0.91
		1	0.77	0.9	6	0.93	0.47	173.31	0.91
		1.5	0.74	0.95	6	0.95	0.49	179.4	0.91
		2	0.73	0.92	5	0.92	0.5	181.12	0.91
	**Best**	2	0.71	1.03	6	0.94	0.44	212.63	0.96
**AM1BCC**	**First**	0.5	0.65	1.12	6	0.9	0.56	117.99	0.87
		1	0.64	1.13	6	0.9	0.56	118.9	0.88
		1.5	0.64	0.96	6	0.94	0.57	116.85	0.88
		2	0.74	1.01	6	0.93	0.42	217.07	0.9
	**Best**	2	0.72	0.94	6	0.92	0.49	173.85	0.9
**MMFF94**	**First**	0.5	0.74	0.94	5	0.92	0.51	175.71	0.92
		1	0.74	0.95	5	0.92	0.51	176.35	0.91
		1.5	0.72	0.98	5	0.92	0.51	172.22	0.91
		2	0.78	0.88	5	0.95	0.39	263.64	0.93
	**Best**	2	0.74	0.99	6	0.95	0.42	240.75	0.96
**Structure-Based Method**									
**MMFF94**	**Best**	2	0.682	1.09	6	0.93	0.48	178.46	0.93

Where: ***GS*** grid spacing, **q**^**2**^: cross validated correlation coefficient, ***SEP*** Standard Error of Prediction, ***C*** optimal number of Components, **r**^**2**^: non-cross validated correlation coefficient, ***SEE*** Standard Error of Estimation, ***F*** Fischer test values, $r_{\mathrm{pred}}^{2}$: prediction of external test set for validation.

**Table 4:** Ligand-based and structure-based CoMSIA models along with percentage contribution of their descriptors

**COMBINATIONS**	**q**^**2**^	**r**^**2**^	$r_{\mathrm{pred}}^{2}$	**F**	**C**	**SEE**	**SEP**	**1%**	**2%**	**3%**	**4%**	**5%**
S+ES	0.714	0.927		150.91	6	0.524	1.038	37.1	62.9	_	_	_
S+H	0.728	0.935		171.61	6	0.493	1.014	42.9	57.1	_	_	_
S+D	0.681	0.89		95.284	6	0.645	1.096	62.5	37.5	_	_	_
S+A	0.756	0.941		190.33	6	0.47	0.959	55	45	_	_	_
S+D+A	0.727	0.936		171.69	6	0.493	1.015	42	34.7	23.3	_	_
ES+H	0.728	0.935		171.28	6	0.494	1.014	53.2	46.8	_	_	_
ES+D	0.69	0.912		122.84	6	0.576	1.082	70.6	29.4	_	_	_
ES+A	0.755	0.945		203.06	6	0.456	0.96	62.1	37.9	_	_	_
ES+D+A	0.713	0.938		178.42	6	0.484	1.041	50.8	28.8	20.4	_	_
H+D	0.692	0.917		130.07	6	0.561	1.078	68.2	31.8	_	_	_
H+A	0.745	0.942		193.39	6	0.466	0.98	59.7	40.3	_	_	_
H+S+ES	0.731	0.946		206.42	6	0.452	1.007	35.1	24.8	40.2	_	_
H+D+A	0.717	0.934		167.58	6	0.499	1.033	47	30.9	22.1	_	_
D+A	0.493	0.825		55.777	6	0.812	1.383	41	59	_	_	_
D+S+ES	0.707	0.932		161.84	6	0.507	1.051	23.3	27.7	49	_	_
A+S+ES	0.755	0.952		232.8	6	0.427	0.962	29.1	26.9	44	_	_
S+ES+D+A	0.732	0.948		214.79	6	0.444	1.005	21.9	37.7	22.6	17.8	_
S+H+D	0.718	0.936		173.05	6	0.491	1.032	32.7	43.8	23.5	_	_
S+H+A	0.766	0.952		234.85	6	0.425	0.939	30.2	40	29.9	_	_
ES+H+D	0.727	0.94		185.88	6	0.475	1.014	44	35	21	_	_
ES+H+A	0.761	0.953		238.73	6	0.422	0.949	40.5	33.2	26.3	_	_
H+D+S+ES	0.733	0.948		216.44	6	0.442	1.003	28	18.6	19.8	33.6	_
H+A+S+ES	0.762	0.958		272.47	6	0.396	0.948	27.2	21.5	19.5	31.8	_
S+H+D+A	0.747	0.949		220.3	6	0.438	0.977	25.1	33.6	23.5	17.8	_
ES+H+D+A	0.747	0.954		246.39	6	0.416	0.977	34.9	27.4	21.4	16.2	_
**H+S+ES+D+A**	**0.751**	**0.958**	**0.93**	**270.35**	**6**	**0.398**	**0.97**	**22.8**	**16.5**	**28.2**	**18.1**	**14.4**
**Structure Based Model**												
**H+S+ES+D+A**	**0.664**	**0.955**	**0.927**	**252.32**	**6**	**0.411**	**1.125**	**26.2**	**15.4**	**25.4**	**16.7**	**16.3**

Where: **q**^**2**^: cross validated correlation coefficient, **r**^**2**^: non-cross validated correlation coefficient, $r_{\mathrm{pred}}^{2}$: prediction of external test set for validation, ***F*** Fischer test values, ***C*** optimal number of Components, ***SEE*** Standard Error of Estimation, ***SEP*** Standard Error of Prediction, **%1-5**: percentage contribution of descriptors in the field, respectively, ***S*** Steric field, ***ES*** Electrostatic field, ***H*** Hydrophobic descriptor, ***D*** hydrogen bond Donor field, and ***A*** hydrogen bond Acceptor field.

### Statistics of the receptor based models

In ligand-based approach, several combinations of charges and grid spacing were used. Among them, the model generated by using MMFF94 charges was retrieved as the best model with q^2^ value of 0.74, standard error of prediction was 0.99 and the r^2^ value of 0.96. The results are summarized in Table 4. For structure-based method, the bound conformation of Darunavir in the crystal structure of HIV protease (PDB: 3QOZ) [17,18] was used as a template to align the series of 102 compounds (Figure 1c). As shown in Table 1, the structure-based QSAR method returned with the q^2^ value of 0.682, r^2^ of 0.938, F value of 178.46 and lower standard error of estimate and standard error of prediction with an average residual values of 0.077. While the r^2^ value of the test set was 0.947. This statistical evaluation showed that the performance of the structure-based method was comparable to the ligand-based approach for CoMFA studies (Table 3).

In CoMSIA, cross validated value of 0.664 and 0.751 was obtained for the structure-based and ligand-based methods, respectively. The CoMSIA analysis is tabulated in Table 4. Similarly, predictive r^2^ value was 0.927 and 0.929 for structure-based and ligand-based methods, respectively.

### Contour maps of CoMFA

CoMFA contours of different colors represented different fields i.e. steric (bulky favored- green whereas yellow is indicative of bulky disfavored area). Similarly, blue and red regions described electron donating and accepting groups would be favored or disfavored, respectively.

**Figure 2:**
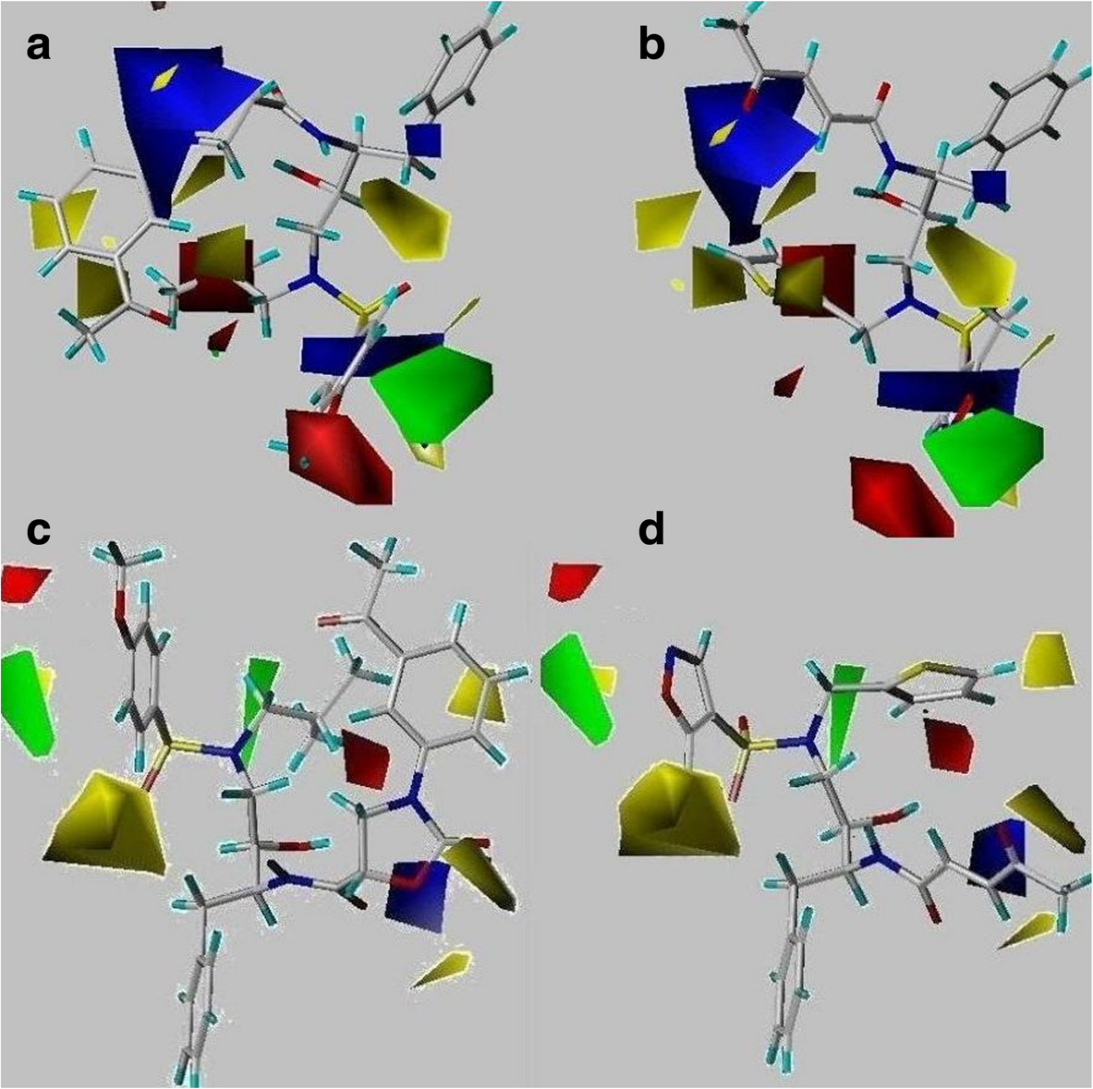
**CoMFA contour maps.** The contour maps of CoMFA modeling, sterically favored areas are represented by green isopleths while yellow regions are served for sterically unfavorable regions. However, electropositivity and electronegativity are represented by blue and red contours, respectively. **a**-**b** are representative of ligand-based CoMFA descriptors of most active (comp-32) and least active (comp-46) whereas **c**-**d** demonstrate structure-based CoMFA contour maps with active and in-active compounds, 32 and 46, respectively.

Figure 2a and 2c displayed CoMFA generated steric and electrostatic contour maps for ligand-based and structure-based models, respectively. The most active compound 32 was superimposed on the steric and electrostatic contours maps for clear illustration.

The analysis of contour maps generated by ligand and structure-based methods showed that the electronegativity (red polyhedral) is favored at R_1_ position in compound 32 where 3-phenyloxaolidin-2-one ring is present. While, the presence of prop-1-ene group at this position in compound 46 has a negative effect on the biological activity depicted in Figure 2b (ligand-based) and 2d (structure-based). Similarly, electropositivity (blue contours) is favored between benzene ring and nitrogen of 3-phenyloxaolidin-2-one in compound 32. The increase or decrease in electronegativity, represented by red contours at R_1_, indicated its effect on observed biological activities. If we compared compounds 28–31 with 7–14, it was found that they have huge difference in their inhibitory activity due to the difference in number of electronegative fluorine at R_1_ position which buried near red isopleth. Even the compounds having propanone moiety at same position, more declined activity was observed. Second red polyhedral was observed near R_2_ position, surrounded the isobutane moiety of compound 32, which demonstrated that the substitution of electronegative element at this position could further enhance the biological activity of the compound 32.

At R_2_ position, less bulky group would be favorable for biological activity, indicated by yellow polyhedral. Compounds 4, 6, 21 and 55 contained bulky group at this position and considered as less effective with inhibitory activity as compared to active. Similarly, comparison of compound 43 with template 32, it was revealed that replacement of 2-methyl thiophene with less bulky substituent at R_2_ position would help to enhance its inhibitory activity. A large green polyhedral found near R_3_ position indicating if replaced anisole moiety of compound 32 with more bulkier group would be beneficial for better activity.

Presence of methoxy phenyl at *para* position of compound 32, strongly favored the inhibitory activity as electronegative and bulky group is required at R_3_ position. Compounds which pose methoxy phenyl group at this position, showed activity not less than 8.38. While compounds 43 and 46 contained isoxazole group at this position, could be the reason of their reduced activity.

### Contour maps of CoMSIA

The CoMSIA steric and electrostatic descriptors were found to be identical with the CoMFA generated models, which proved the consistency of the results. Moreover, the results of other three descriptors of CoMSIA also improved the drug prediction. The hydrogen bond donor and acceptor descriptors revealed the reason of higher activity of compound 32. At R_1_ position of 32, the purple polyhedral is surrounded which showed that this is donor disfavored region. In compound 32, this donor disfavored region is supplemented by the presence of highly electronegative elements in 3-phenyloxazolidin-2-one ring. At R_2_ position hydrogen bond acceptor is disfavored (red polyhedral); at this position an alkyl chain is present in compound 32. At R_3_ position a hydrogen bond acceptor is favored (magenta polyhedral), which is supplemented by the presence of methoxy group. In contrast, these properties are absent in least active compound 46 which possibly the reason of its lower activity.

**Figure 3:**
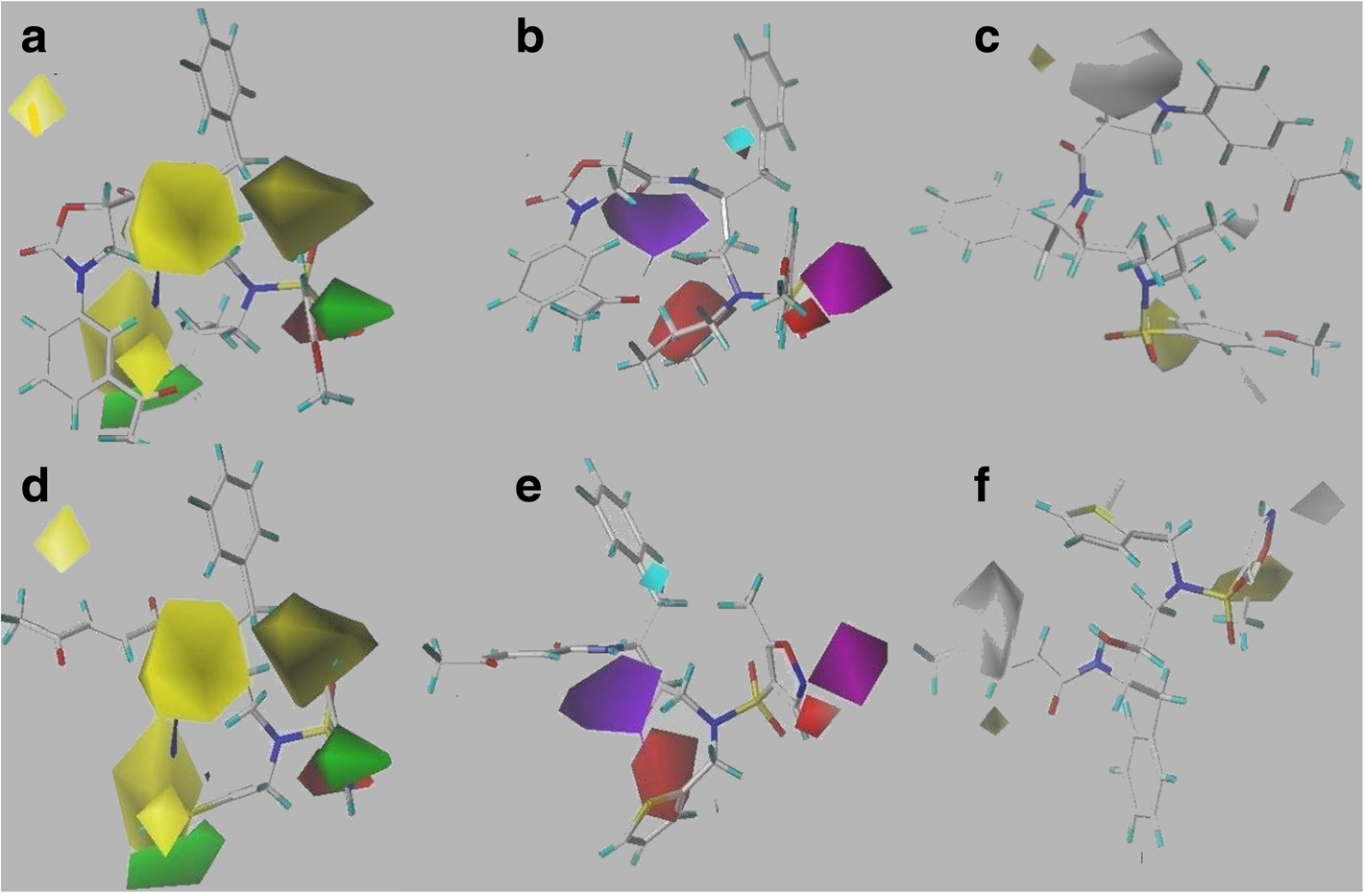
**CoMSIA ligand-based descriptors.** Representation of ligand-based CoMSIA descriptors with most active and least active compounds. **a**-**c** depicted steric & electrostatic, acceptor & donor and hydrophobic descriptor maps of most active compound, respectively (32), whereas **d**-**f** showed all five descriptor contours with least active compound (46).

**Figure 4:**
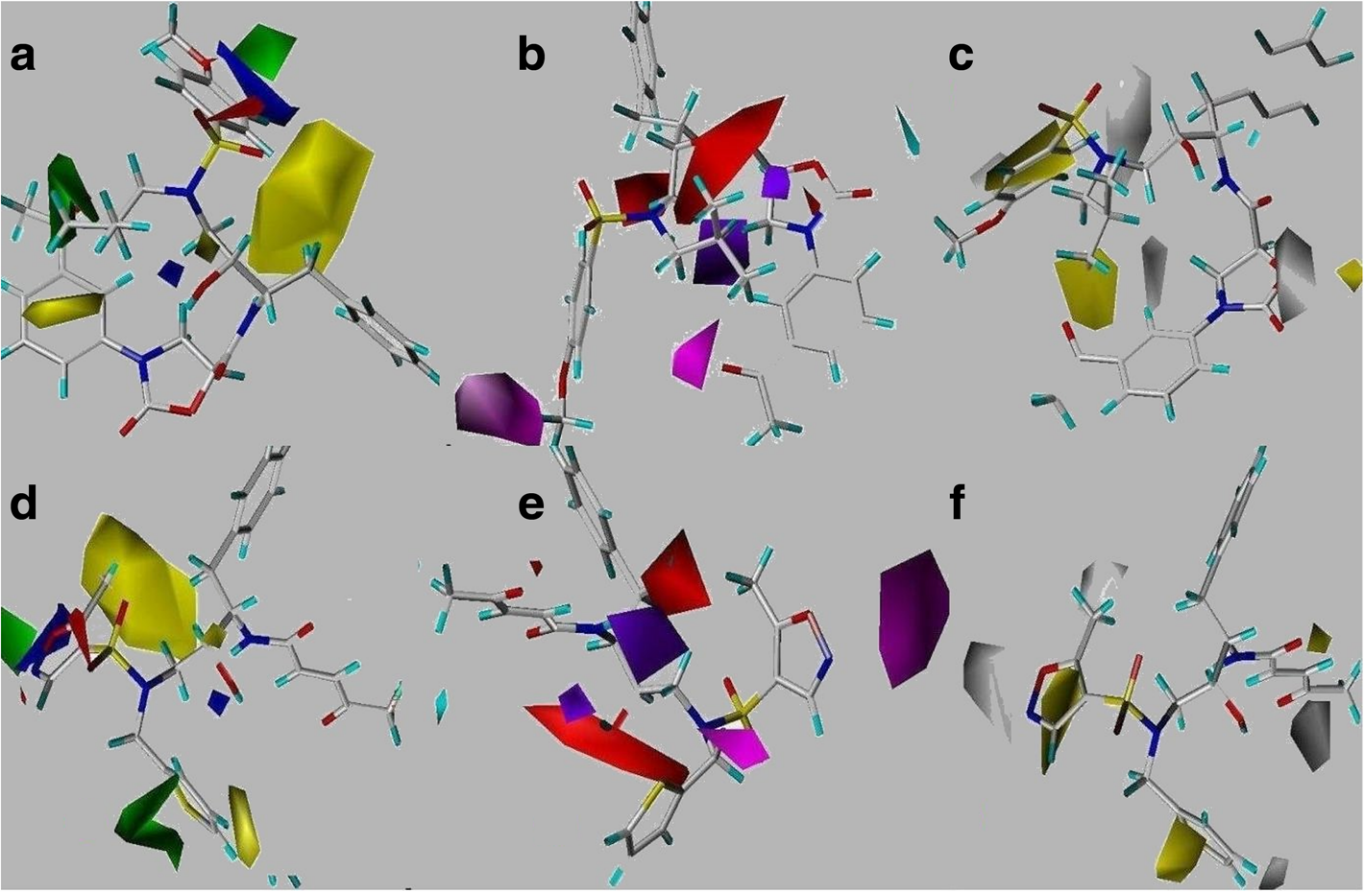
**CoMSIA structure-based maps.** Illustration of structure-based CoMSIA descriptors. Upper portion marked as **a**-**c** displayed steric & electrostatic, acceptor & donor as well as hydrophobic contour maps of compound 32 claimed as most active. However, **d**-**f** are representative of compound 46’s descriptor maps marked as least active compound within the series.

The hydrophobic descriptor of CoMSIA is important to evaluate the hydrophobicity required to sustain the biological activity of any compound. At R_1_ position, hydrophobicity is highly disfavored (white isopleth) whereas R_3_ is hydrophobic favored (yellow contours) region. As shown in Figure 3c, compound 32 contained nitrogen containing hydrophilic moiety at R_1_ position while this hydrophilic moiety is absent in compound 46 (Figure 3f). In compound 32, the R_3_ position is substituted with the phenyl-methoxy group while compound 46 contained hetero-atomic methyl-isoxazole moiety at R_3_ position, showed that hydrophilic substitution at R_3_ position would decrease the biological activity of compound 46. The CoMSIA contour maps of compound 32 and 46 with ligand-based and structure-based approaches are presented in Figures 3 and 4, respectively.

### Docking results

**Figure 5:**
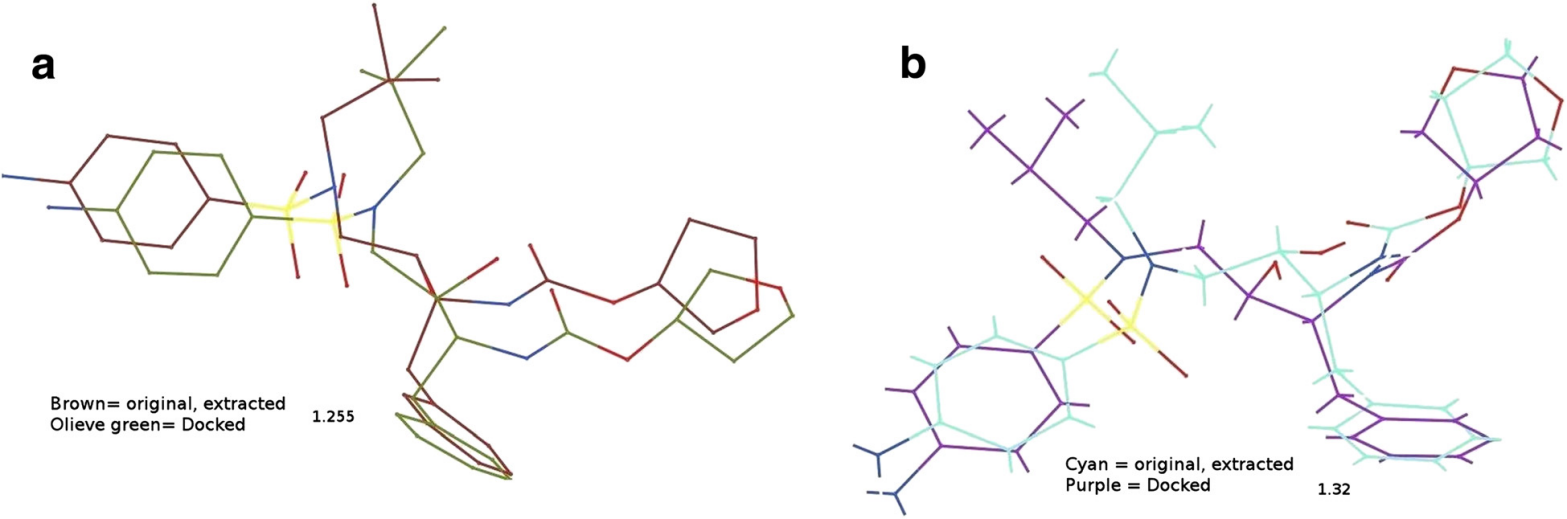
**Re-docking poses and RMSD values.** Re-docking results of **a**) wild type (3EKV) and **b**) mutated (3NU9) proteins with RMSD of 1.255Å and 1.32Å, respectively.

To validate the 3D-QSAR results, docking simulation was performed and the most active compound 32 and least active compound 46 was evaluated for their binding interactions in the active site of protease and results were compared. Initially, the performance of docking software was tested by re-docking experiment. For this purpose, crystal structures of two proteins with their cognate ligands were retrieved from PDB and the cognate ligands were re-docked. The results are summarized in Table 5. The superimposed view of docked conformation and the reference ligand is presented in Figure 5a-b. Based on the re-docking results, GOLD was used for docking. The comparison of the scores attributed by two scoring functions as Gold-Score and Chem-Score also showed the compound 32 to be more active than 46 in both wild and mutated proteins. However, Gold-score showed drastic difference between the scores of two compounds which can be assumed on this basis to more accurate than Chem-Score.

**Table 5:** Re-docking and docking results of wild type and mutated with most active and least active compounds

**PDB ID**	**Resolution**	**Type**	**RMSD**
3EKV	1.75	Wild	1.36
3NU9	1.85	Mutated	1.22
**Docking of most and least potent compounds**			
**Docked Comp.**	**PDB ID**	**Gold-Score**	**Chem-Score**
32 (Highly active)	3EKV	81.63	23.86
	3NU9	72.54	22.2
46 (Least active)	3EKV	78.11	14.82
	3NU9	55.68	14.1

**Figure 6:**
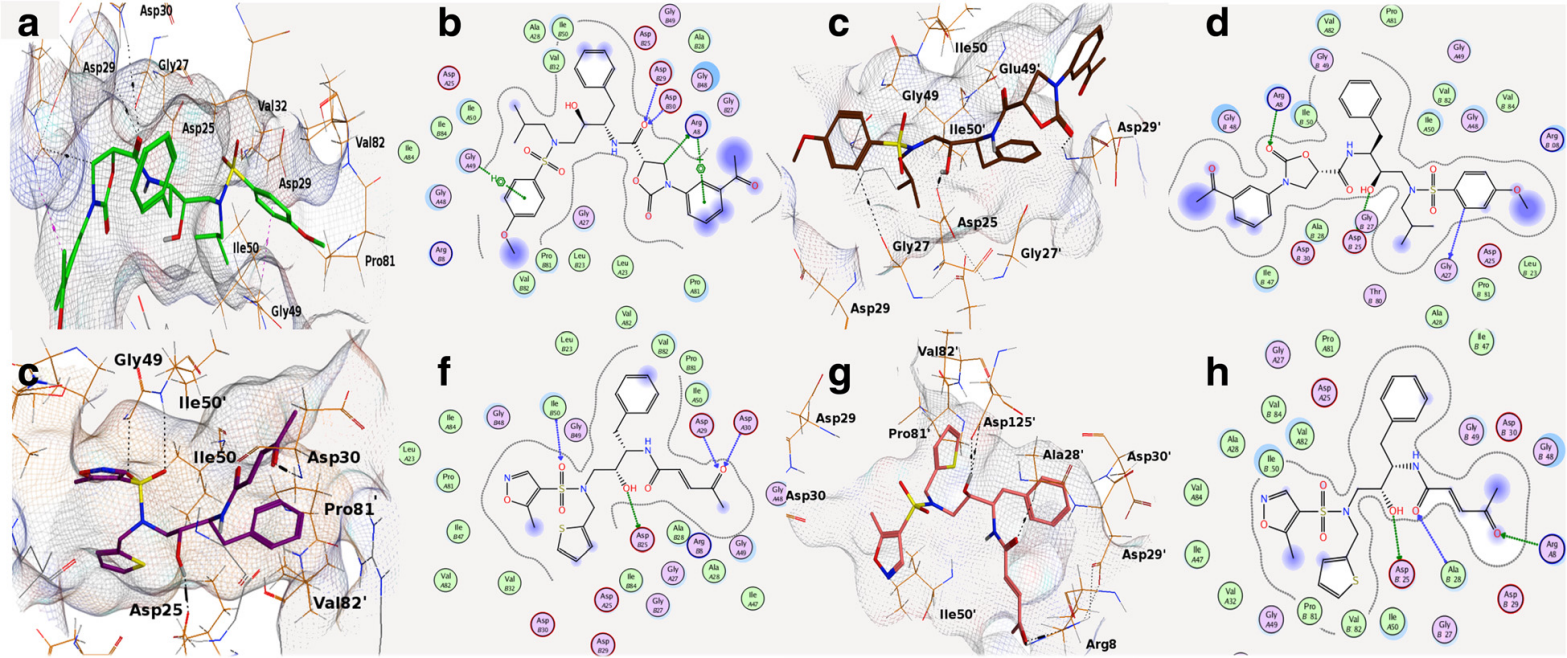
**2D and 3D docking representations.** A representation of docking interactions and poses of most active and least active molecules with wild type and mutated HIV-protease protein via 2D and 3D representations. **a**-**d** most active compound (32) interacted with important active site residues of wild type and mutated proteins, respectively. Similarly, **e**-**h** represents interactions of least active compound within binding pocket of wild type and mutated proteins to show how the compound 46 lost its interactions and activity due to conformational change occurred in response to mutation.

On the basis of docking analyses, it was revealed that compound with highest activity (32) ranked at top position as compared to least active compound 46. The docking scores were in correlation with 3D-QSAR and experimental results. The docked conformation of compound 32 in wild type (Figure 6a-b) and mutated proteins (Figure 6c-d) revealed that compound interacted with the binding pocket residues of targeted proteins through several favorable interactions including polar, hydrophobic, hydrogen bonding and the weak Van der Waal contacts.

The carbonyl oxygen of the core structure near R_1_ position also mediated strong hydrogen bonding with the backbone amino group of Asp29' and Asp30'. Moreover, hydrophobic interactions were observed between Ile50 and core group of compound 32 and acetophenone with Arg8. Pro81' also mediated hydrophobic interaction with the methyl group of methoxybenzene present at R_3_ position. Furthermore side-chains of S_1_' residue Val82' mediated CH--π contact with the hydrophobic portion of the ligand at R_3_ position. Val32' mediated CH_3_--π interactions with the core benzene of compound 32.

The observed docked conformation of compound 32 in the mutated protein (I84V) was flipped at ~90°, showed in Figure 6c-d. Even with this orientation, the ligand was found to be interacting with several important residues including Gly27, Gly27', Asp25, Asp25', Asp29, Asp29', Ile50, GLy49' and Ile50'. In this case the Gly27 interacted with R_2_ substitution and Gly27' with core structure of compound 32. A hydrogen bond was observed between the side chain oxygen of Asp25' and the hydroxyl of compound 32 (2.04Å). Furthermore Asp29' mediated a strong hydrogen bond with oxygen atom of R_1_ 3-phenyloxazolidin-2-one ring with the distance of 1.95Å. Moreover, the compound is stabilized by the hydrophobic interactions offered by Ile50, Gly49' and Ile50'. These interaction patterns of compound 32 with the wild type and mutated forms of protein suggested that the modification at R_2_ position could increase the activity of compound. This hypothesis further confirms the results obtained by CoMFA.

The docked conformation of compound 46 in the wild type protein (Figure 6e-f) revealed that it formed CH_3_--π interaction with side chain of Ile50 and Val82', however, core benzene of compound 46 also mediated aromatic interaction with Pro81'. On the other hand, Asp25 interacted with hydroxyl oxygen of core structure whereas Ile50’ attracted towards oxygen of sulfonamide near R_3_ substituent.

The terminal methoxy oxygen at R_1_ mediated interactions with the wild type protein’s amino group of Asp29 and Asp30 with the distance of 2.29Å and 1.7Å, respectively. The interactions of compound 46 with these residues were lost upon mutation (Figure 6g-h). The binding orientations of compound 32 and 46 (Figure 6) revealed that compound 32 maintained its interactions with the active site residues in wild type as well as in mutated protein while compound 46 lost most of its binding interactions in mutated protein as shown in Table 6.

**Table 6:** Protein-ligand binding interactions with specific conserved residues

**Res.**	**Spec**	**Compound 32**				**Compound 46**			
		**W.T**	**Bonds**	**I84V**	**Bonds**	**W.T**	**Bonds**	**I84V**	**Bonds**
**Gly**	**27**			√	HC…π			√	O…HC
	**27'**	√	CH…O (2.01Å)	√	O…HC				
	**48**								
	**48'**	√	HO…HC						
	**49**								
	**49'**			√	CH…O				
**Asp**	**25**								
	**25'**		OH…O (2.04Å)	√	OH…O				
	**29**				O…NH	√	O…NH (2.29Å)		
	**29'**	√	CO…NH (1.95Å)	√					
	**30**					√	O…NH (1.75Å)		
	**30'**	√	CO…NH						
**Val**	**82**								
	**82'**	√	CH…π	√	CH…O	√	CH…π	√	HC…CH
	**32**								
	**32'**	√	CH…π			√	CH…HC		
**Pro**	**81'**	√	CH…π			√	C-H…O	√	HC…π
**Ile**	**50**	√	CH…CH						
	**50'**			√	NH…π			√	CH…O
	**84'**						CH…π		

**Note:** Distances of important interactions are shown in Å, however, all interactions mentioned here having distances of less than 3Å.

## Conclusion

In the present work, comparison of ligand and structure-based 3D-QSAR using CoMFA and CoMSIA were derived for HIV-1 protease inhibitors. The statistics of both models were convincing and comparable. The model was significantly favored by internal and external predictions as well as visualization of contour maps. The effect of important structural characteristic of the potent inhibitor was predicted by the generated model. From the predictions, it was evident that at R_1_ position electronegativity is favored due to presence of Asp29 in its vicinity and hydrophobicity is disfavored which is relevant with the presence of methyloxazolidione ring in compound 32. Docking results also showed that terminal methoxy oxygen at R_1_ mediated bidentate interactions with the amino group of Asp29 and Asp30 which was lost in compound 46. At R_2_ position, bulkiness is disfavored whereas at R_3_; hydrophobicity is favored which is evident by presence of methoxy phenyl in compound 32. The docking studies of most potent and least active inhibitors further verified the generated 3D-QSAR models and can be used as guidance for better drug development.

## Methodology

### Dataset preparation

The dataset of 102 compounds was retrieved from literature reported by *Jorissen R.N. et al.*, [7] and available in Additional file 1. 2D *s*tructures were drawn by Chem-Draw [19] and converted into 3D by MOE (Molecular Operating Environment) program [20]. The biological activities of all compounds were shown in Table 1 along with its negative logarithmic units, pIC_50_ values. Stereochemistry and atom typing were confirmed for each compound. Three different charges i.e., GH, AM1BCC and MMFF94 were applied to the dataset and all three sets were subjected to the database alignment by using sybyl7.3 [16]. The database alignment is depicted in Figure 1. The core structure of most active compound 32 (pIC_50_ = 12.10) was used as a template for alignment [21] in ligand-based QSAR. On the other hand, for structure-based QSAR, bound conformation of original compound was used as template for alignment.

### CoMFA & CoMSIA 3D-QSAR models

The dataset of 102 compounds were segregated into training and test sets containing 78 and 24 compounds, respectively (Tables 1 and 2). Each set was constructed on basis of regular distribution of biological activities (Table 1). Comparative Molecular Field Analysis (CoMFA) and Comparative Molecular Similarity Indices Analysis (CoMSIA) with 2Å grid spacing, sp^3^ carbon probe atom with a charge of +1 and VdW radius of 1.52Å was used to calculate steric and electrostatic field descriptors. In order to reduce noise and improve efficiency, column filtering of 2.0 kcal mol^-1^ was used [16]. A default cutoff of 30 kcal mol^-1^ was used for field energy calculations. Subsequently partial least square (PLS) analysis was performed to obtain 3D-QSAR model.

The optimal number of components was determined by leave-one-out procedure (Cross validation) to build the statistical significant regression model. The quality of the model was judged by cross-validated coefficient q^2^ which should not be less than 0.5. The external predictivity was calculated by conventional correlation coefficient r^2^[22,23].

### Molecular docking by GOLD

The dataset of 102 compounds was subjected to docking in order to validate the QSAR results via GOLD docking suit [12]. The emphasis was totally on most active and the least active compounds to evaluate their quality of interaction as HIV-1 protease inhibitors. For docking, wild type (PDB: 3EKV) [24], and mutated I84V (PDB: 3NU9) [25] proteins were retrieved from Protein Data Bank (PDB) [26] in order to check the consistency of ligand’s interactions even if mutated viral attack is present.

The cognate ligand and water molecules were removed, and polar hydrogens were added. Software was validated by re-docking and root mean square deviation (RMSD) calculations shown in Table 5 and Figure 5. Default GOLD docking parameters were used with Gold-score and Chem-score as scoring and rescoring functions. For each ligand, ten docked poses were saved and analyzed.

## Abbreviations

3D-QSAR: 3-dimentional quantitative structure-activity relationship; HIV: Human immunodeficiency virus; AIDs: Acquired immunodeficiency syndrome; FDA: Food and drug administration; HAART: Highly active antiretroviral therapy; RT: Reverse transcriptase; PIs: Protease inhibitors; IN: Integrase; DRV: Darunavir; CoMFA: Comparative molecular field analysis; CoMSIA: Comparative molecular similarity index analysis; GOLD: Genetic optimization for ligand docking; MOE: Molecular operating environment; PLS: Partial least square; RMSD: Root mean square deviation.

## Competing interests

The authors declared no competing interests.

## Authors’ contributions

ZQ supervised, conceived and guided the whole project and the manuscript. SU and HS carried out the work and drafted the manuscript with UM and SAH. All authors have read and approved the final manuscript.

## Supplementary Material

Additional file 1 **Darunavir derivatives with all sibstitutions.** Core structure of darunavir with positions marked for substitutions and structures of substituents at R_1_, R_2_ and R_3_ positions along with their experimental inhibitory activities. Click here for file
