# Supplementary material for: A combined 3D-QSAR and docking studies for the In-silico prediction of HIV-protease inhibitors
Source: Chem Cent J. 2013 May 17;7:88. doi: 10.1186/1752-153X-7-88 (PMC3660290; doi:10.1186/1752-153X-7-88)
Supplement: Additional file 1 — Darunavir derivatives with all sibstitutions. Core structure of darunavir with positions marked for substitutions and structures of substituents at R1, R2 and R3 positions along with their experimental inhibitory activities. [file 1752-153X-7-88-S1.pdf]

Additional File-**1**: Darunavir derivatives with all sibstitutions

Core structure of darunavir with positions marked for substitutions and structures of substituents at R_1, R_2 and R_3 positions along with their experimental inhibitory activities.

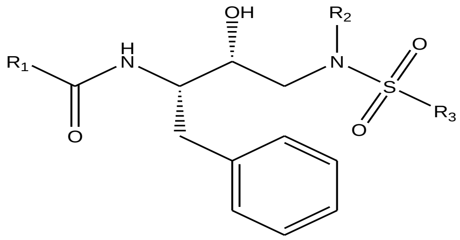

MOLECULES	R_1	R_2	R_3	pK_i
**Compound 01**	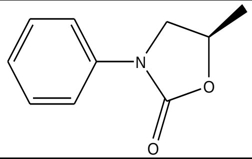	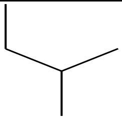	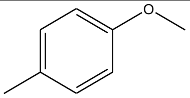	10.00
**Compound 02**	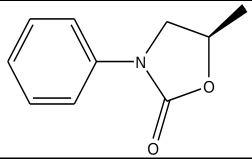	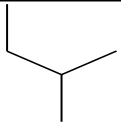	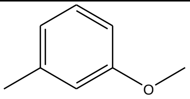	8.42
**Compound 03**	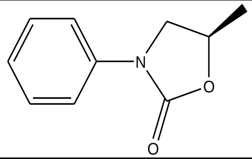	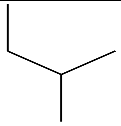	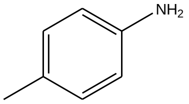	9.28
**Compound 04**	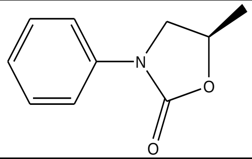	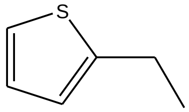	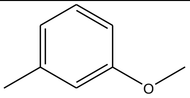	6.62
**Compound 05**	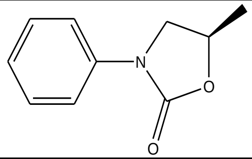	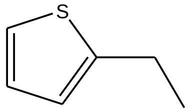	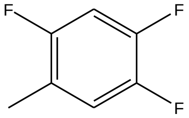	6.77
**Compound 06**	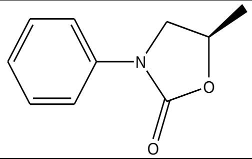	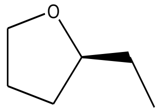	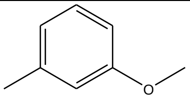	7.38
**Compound 07**	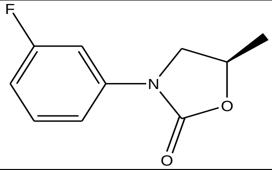	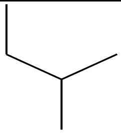	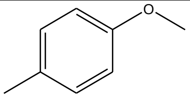	10.08

**Compound 08**	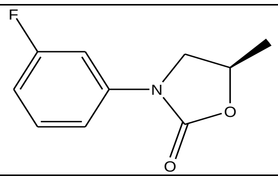	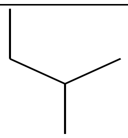	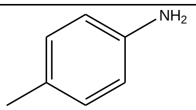	9.77
**Compound 09**	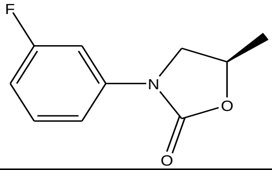	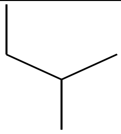	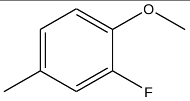	10.15
**Compound 10**	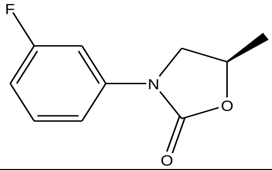	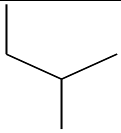	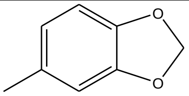	9.97
**Compound 11**	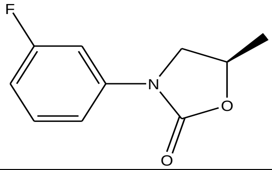	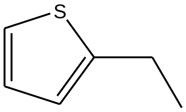	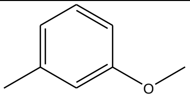	6.72
**Compound 12**	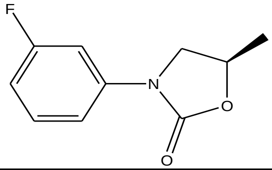	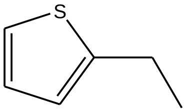	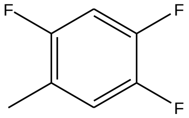	6.80
**Compound 13**	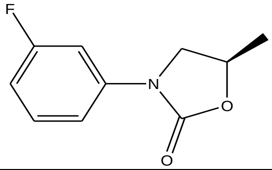	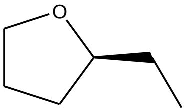	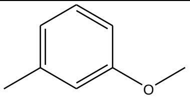	6.82
**Compound 14**	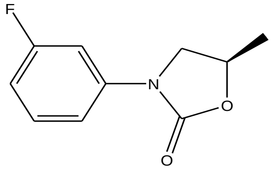	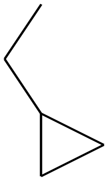	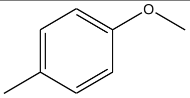	9.59
**Compound 15**	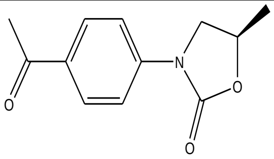	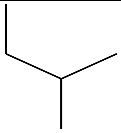	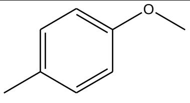	11.40
**Compound 16**	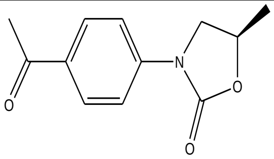	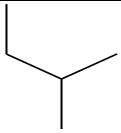	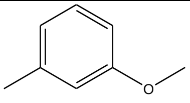	9.08

**Compound 17**	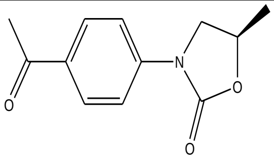	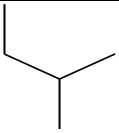	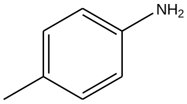	9.74
**Compound 18**	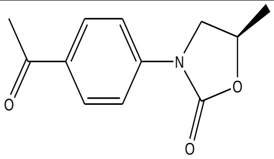	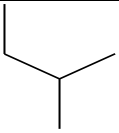	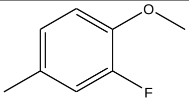	10.10
**Compound 19**	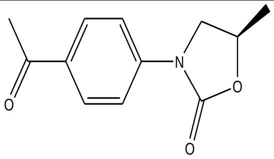	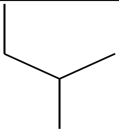	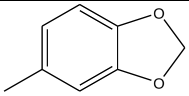	10.80
**Compound 20**	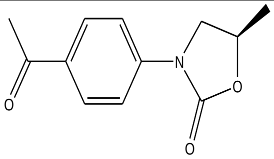	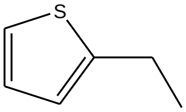	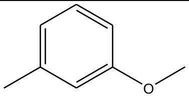	7.53
**Compound 21**	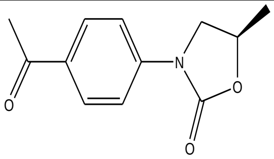	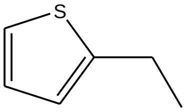	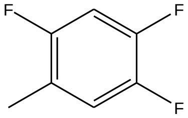	6.78
**Compound 22**	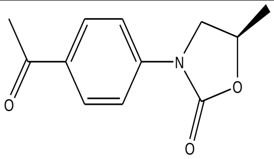	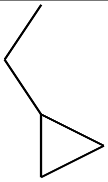	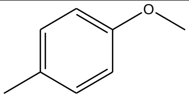	9.10
**Compound 23**	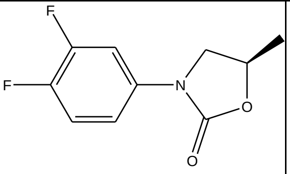	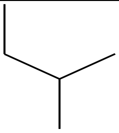	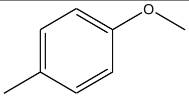	10.18
**Compound 24**	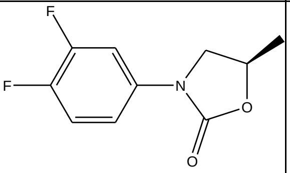	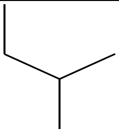	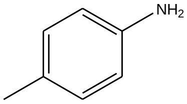	9.64

**Compound 25**	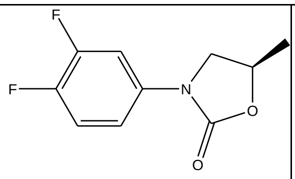	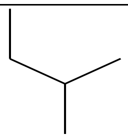	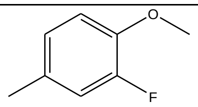	9.46
**Compound 26**	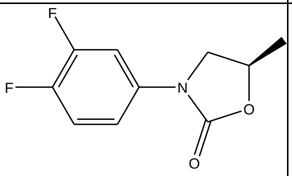	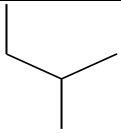	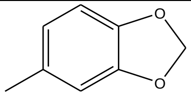	10.07
**Compound 27**	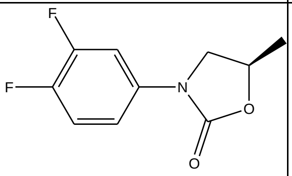	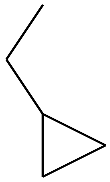	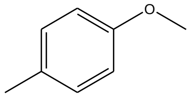	9.24
**Compound 28**	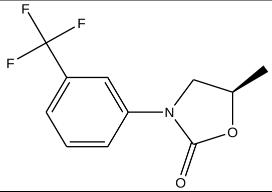	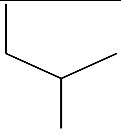	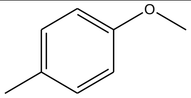	11.22
**Compound 29**	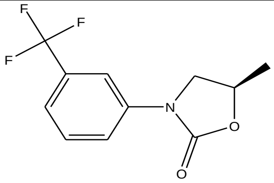	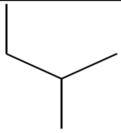	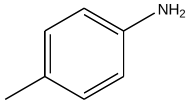	10.38
**Compound 30**	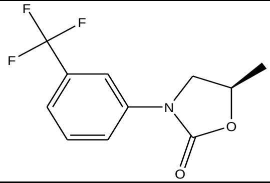	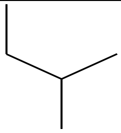	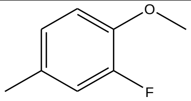	10.14
**Compound 31**	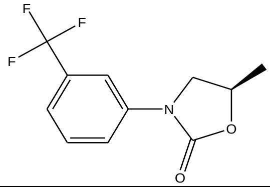	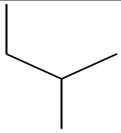	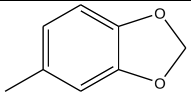	10.80
**Compound 32**	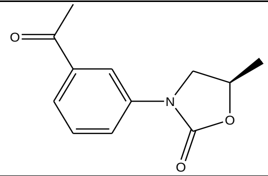	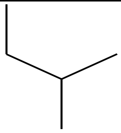	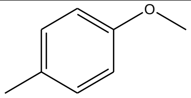	12.10

**Compound 33**	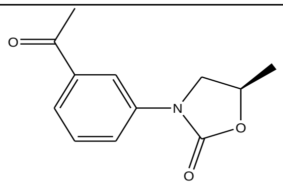	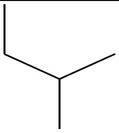	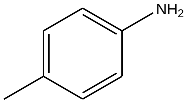	10.49
**Compound 34**	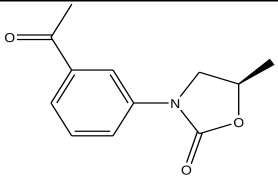	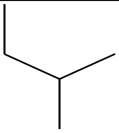	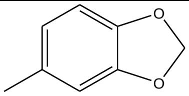	11.22
**Compound 35**	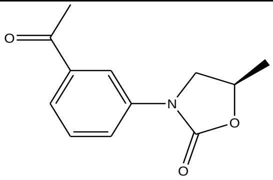	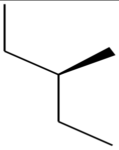	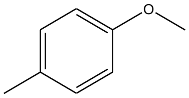	9.63
**Compound 36**	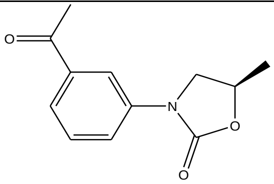	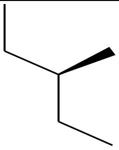	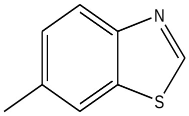	10.72
**Compound 37**	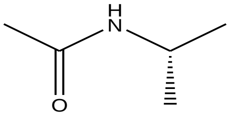	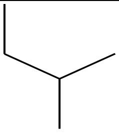	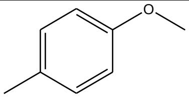	9.93
**Compound 38**	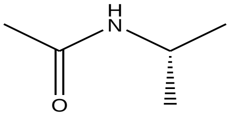	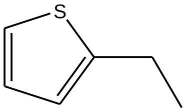	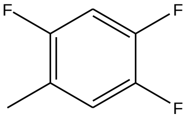	7.48
**Compound 39**	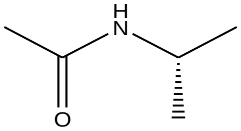	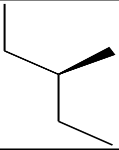	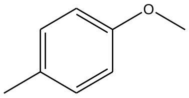	10.34
**Compound 40**	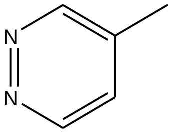	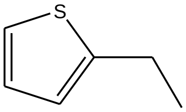	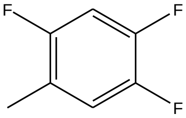	5.97
**Compound 41**	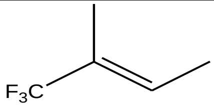	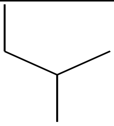	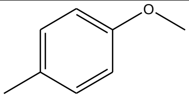	9.41

**Compound 42**	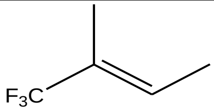	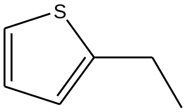	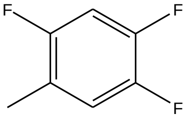	7.28
**Compound 43**	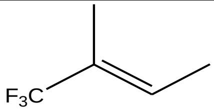	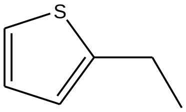	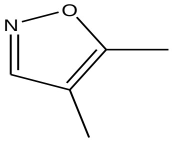	4.88
**Compound 44**	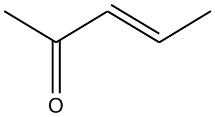	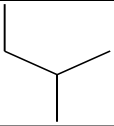	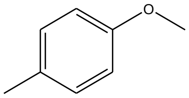	9.77
**Compound 45**	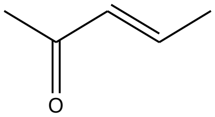	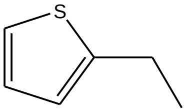	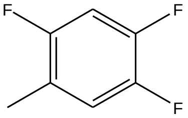	6.21
**Compound 46**	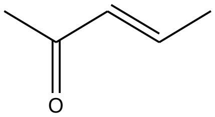	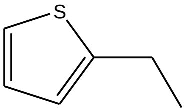	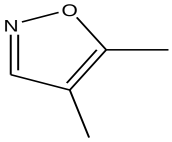	4.58
**Compound 47**	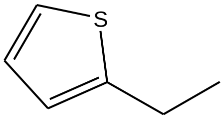	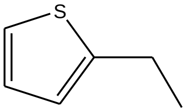	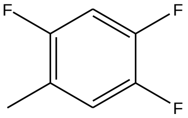	5.63
**Compound 48**	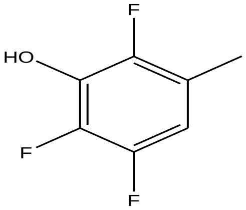	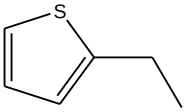	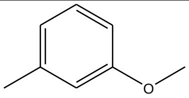	6.29
**Compound 49**	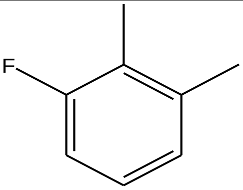	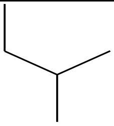	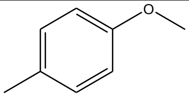	10.03
**Compound 50**	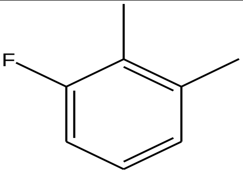	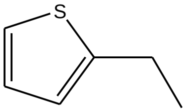	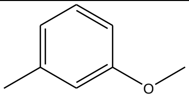	7.39
**Compound 51**	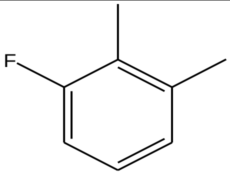	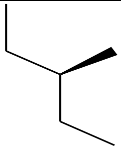	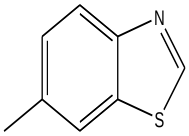	10.48

**Compound 52**	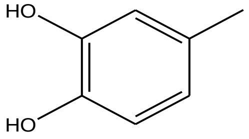	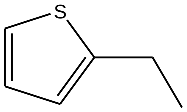	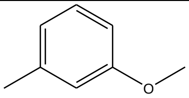	7.30
**Compound 53**	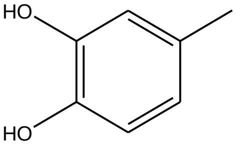	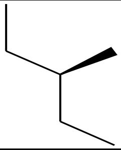	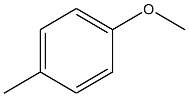	9.42
**Compound 54**	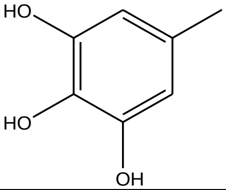	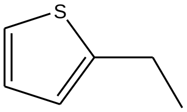	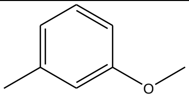	5.94
**Compound 55**	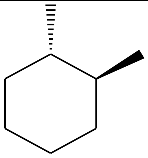	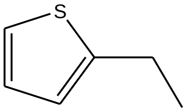	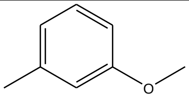	6.23
**Compound 56**	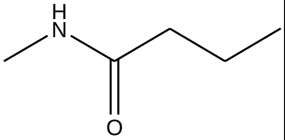	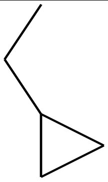	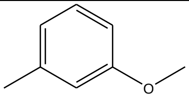	7.62
**Compound 57**	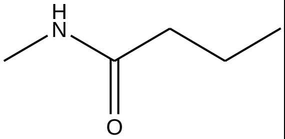	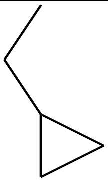	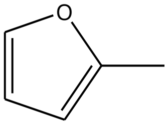	7.24
**Compound 58**	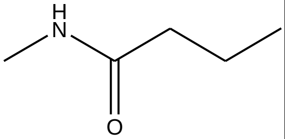	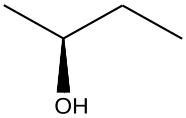	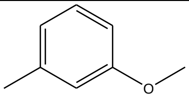	5.93
**Compound 59**	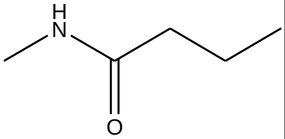	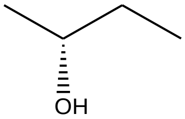	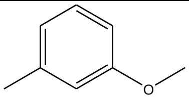	6.12
**Compound 60**	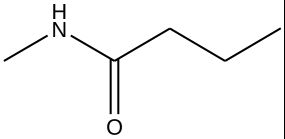	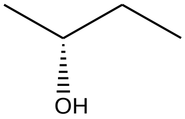	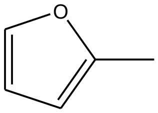	6.27

**Compound 61**	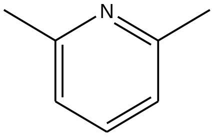	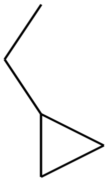	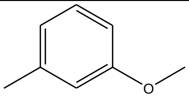	4.84
**Compound 63**	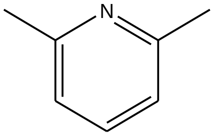	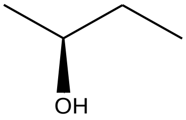	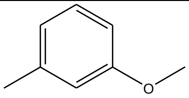	4.91
**Compound 64**	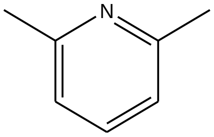	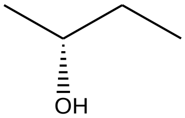	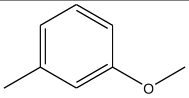	5.32
**Compound 66**	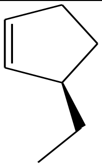	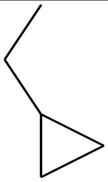	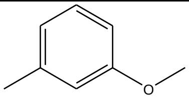	5.69
**Compound 69**	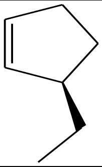	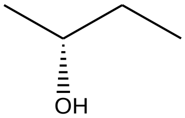	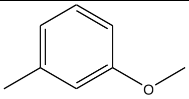	4.87
**Compound 70**	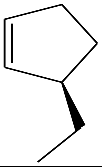	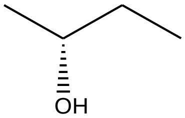	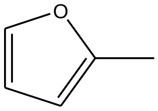	6.59
**Compound 71**	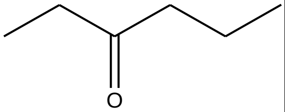	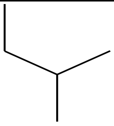	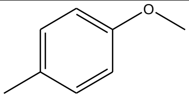	9.62
**Compound 72**	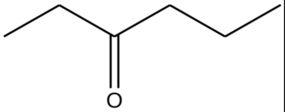	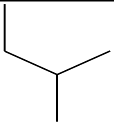	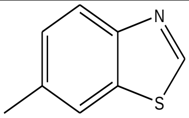	9.92
**Compound 73**	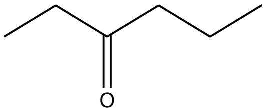	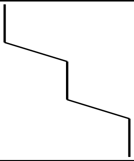	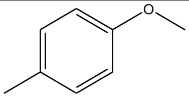	8.38
**Compound 74**	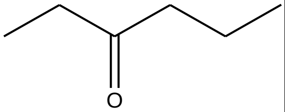	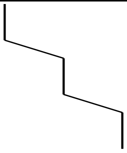	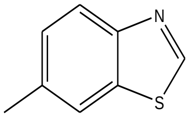	8.79

**Compound 75**	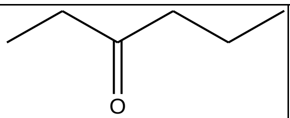	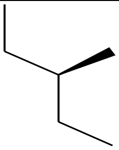	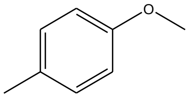	10.21
**Compound 76**	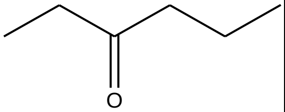	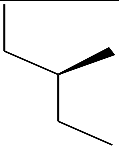	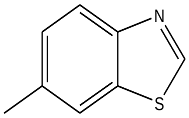	10.20
**Compound 77**	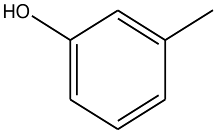	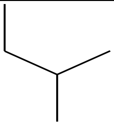	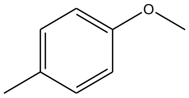	9.85
**Compound 78**	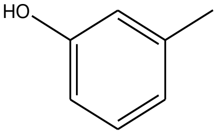	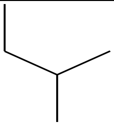	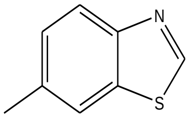	10.57
**Compound 79**	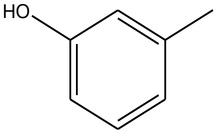	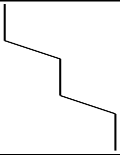	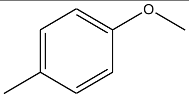	8.84
**Compound 80**	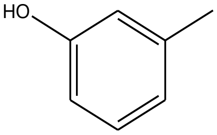	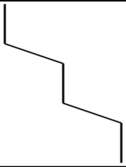	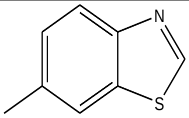	9.51
**Compound 81**	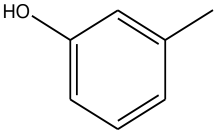	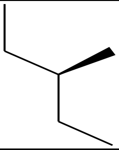	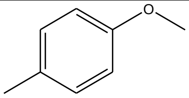	9.93
**Compound 82**	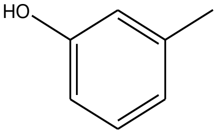	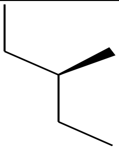	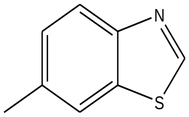	10.44
**Compound 83**	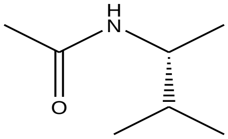	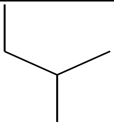	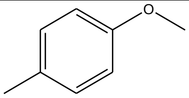	10.08
**Compound 84**	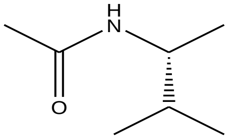	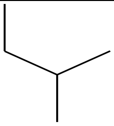	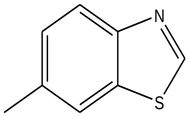	10.00
**Compound 85**	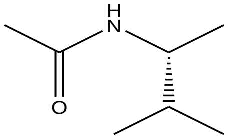	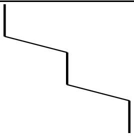	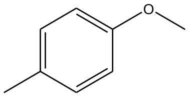	10.42

**Compound 86**	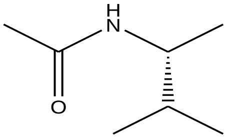	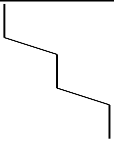	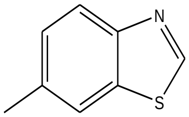	10.85
**Compound 87**	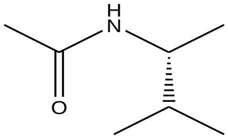	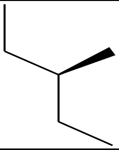	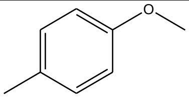	10.48
**Compound 88**	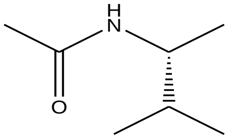	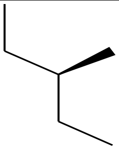	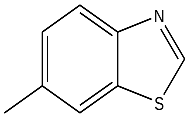	10.24
**Compound 89**	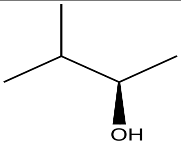	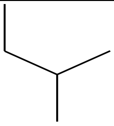	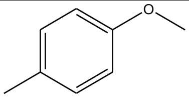	8.73
**Compound 90**	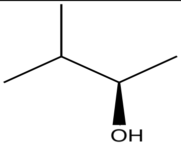	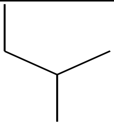	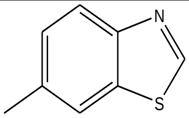	9.54
**Compound 91**	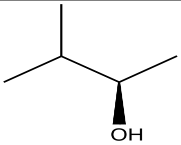	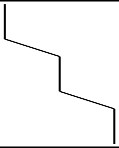	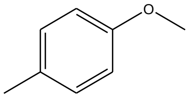	8.61
**Compound 92**	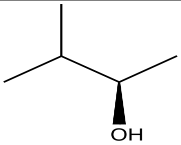	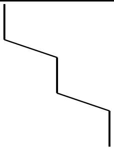	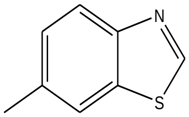	9.09
**Compound 93**	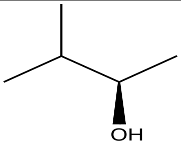	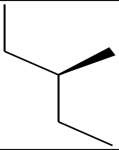	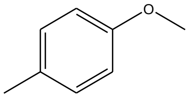	9.21
**Compound 94**	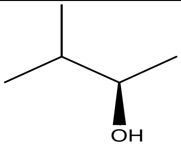	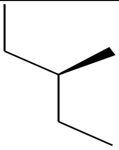	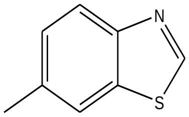	10.20
**Compound 95**	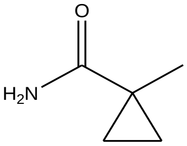	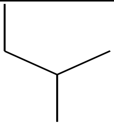	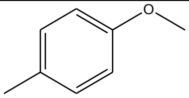	9.76
**Compound 96**	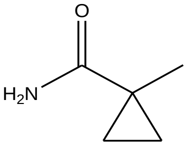	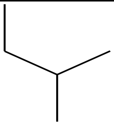	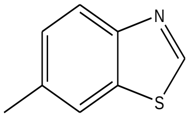	9.94

**Compound 97**	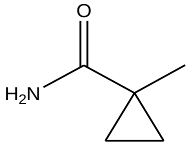	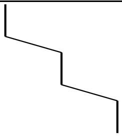	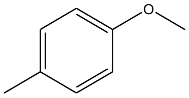	9.10
**Compound 98**	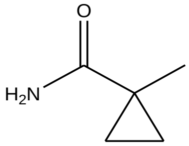	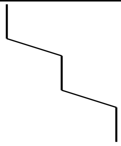	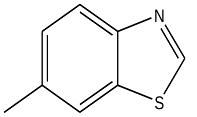	9.68
**Compound 99**	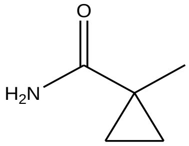	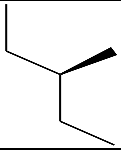	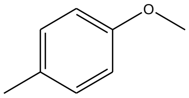	9.68
**Compound 100**	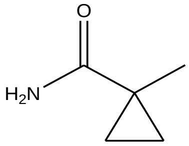	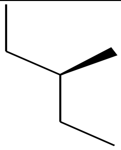	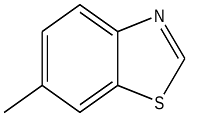	9.88
**Compound 101**	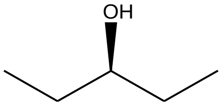	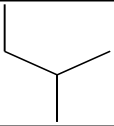	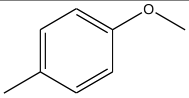	9.43
**Compound 102**	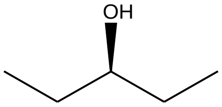	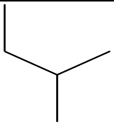	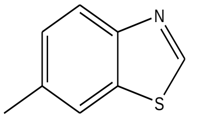	9.87
**Compound 103**	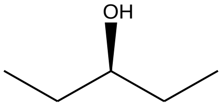	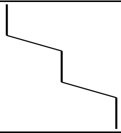	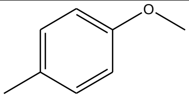	9.07
**Compound 104**	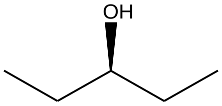	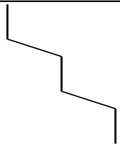	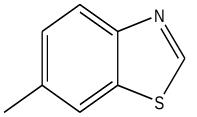	9.15
**Compound 105**	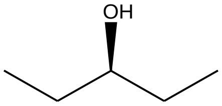	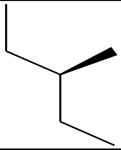	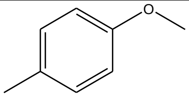	9.47
**Compound 106**	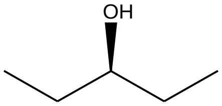	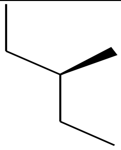	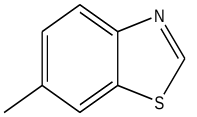	10.17
